# Effects of 405 ± 5-nm LED Illumination on Environmental Stress Tolerance of *Salmonella* Typhimurium in Sliced Beef

**DOI:** 10.3390/foods11020136

**Published:** 2022-01-06

**Authors:** Du Guo, Yichen Bai, Shengyi Fei, Yanpeng Yang, Jiahui Li, Baowei Yang, Xin Lü, Xiaodong Xia, Chao Shi

**Affiliations:** 1 College of Food Science and Engineering, Northwest A&F University, Xianyang 712100, China; duguo2911@nwafu.edu.cn (D.G.); baiyichen_0603@tju.edu.cn (Y.B.); sfei7@huskers.unl.edu (S.F.); yanpengyang@nwafu.edu.cn (Y.Y.); lijiahui@nwafu.edu.cn (J.L.); ybwsheng@nwsuaf.edu.cn (B.Y.); xinlu@nwsuaf.edu.cn (X.L.); meilixinong@nwsuaf.edu.cn (C.S.); 2 National Engineering Research Center of Seafood, School of Food Science and Technology, Dalian Polytechnic University, Dalian 116034, China

**Keywords:** 405 nm LED, *Salmonella* typhimurium, beef slice, environmental stress tolerance, simulated gastric fluid, bile salt

## Abstract

*Salmonella* Typhimurium is a widely distributed foodborne pathogen and is tolerant of various environmental conditions. It can cause intestinal fever, gastroenteritis and bacteremia. The aim of this research was to explore the effect of illumination with 405 nm light-emitting diodes (LEDs) on the resistance of *S*. Typhimurium to environmental stress. Beef slices contaminated with *S*. Typhimurium were illuminated by 405 nm LEDs (18.9 ± 1.4 mW/cm^2^) for 8 h at 4 °C; controls were incubated in darkness at 7 °C. Then, the illuminated or non-illuminated (control) cells were exposed to thermal stress (50, 55, 60 or 65 °C); oxidative stress (0.01% H_2_O_2_ [*v/v*]); acid stress (simulated gastric fluid [SGF] at pH 2 or 3); or bile salts (1%, 2%, or 3% [*w/v*]). *S*. Typhimurium treated by 405 nm LED irradiation showed decreased resistance to thermal stress, osmotic pressure, oxidation, SGF and bile salts. The transcription of eight environmental tolerance-related genes were downregulated by the illumination. Our findings suggest the potential of applying 405 nm LED-illumination technology in the control of pathogens in food processing, production and storage, and in decreasing infection and disease related to *S*. Typhimurium.

## 1. Introduction

*Salmonella*, a Gram-negative bacterium, is a worldwide foodborne pathogen [1]. *Salmonella enterica* has six subspecies, each of which consists of different serovars [2]. Across the world, serovars *S.* Typhimurium and *S.* Enteritidis are recognized as the main etiological agents of salmonellosis in foodborne disease outbreaks [3]. Salmonellosis has four clinical patterns in humans: enteric fever, gastroenteritis, bacteremia, and other complications of nontyphoidal salmonellosis; it also occurs in a chronic-carrier state. The mortality rates of salmonellosis can be as high as 5–7% in some regions. *Salmonella* can multiply in different environments outside living hosts [4]. Foods such as cheese, peanut butter, bean sprouts, egg products and beef can be contaminated by *S.* Typhimurium [5].

Beef is a widely used cooking ingredient in many countries; it can be made into steaks or burgers in different cuisines. Multiple sources of contamination can occur during processing at retail establishments, including chopping, trimming and grinding. Incompletely heated beefburgers are an important source of *Salmonella* infection [6]. Between 2013 and 2017, boneless beef lots and ground beef sublots were produced. *Salmonella* was detected in 1955 boneless beef lots (1.17% of total samples) and 219 ground beef sublots (0.87% of total samples) [7].

During the processes of food production, storage, transportation and sale, contaminating bacteria may face environmental stresses, such as heat, acid or osmotic pressure. *Salmonella* has been reported to survive in environmental stress such as high osmolarity, extreme temperature, and acidic conditions [8]. Moreover, *S.* Typhimurium must face several hurdles after entering the human digestive system, including strong acid conditions and bile salts [3]; the ability of *Salmonella* to survive in such harsh conditions is key to its ability to cause disease.

Recently, light-emitting diodes (LEDs) have been applied for pathogen control and food preservation; this technology is gaining worldwide attention. Previous studies confirmed that visible light in the blue region (i.e., 400–450 nm) can activate photosensitive intracellular porphyrin molecules and generate reactive oxygen species, and thus effectively inactivate microorganisms [9]. A previous study showed that the application of a 405 ± 5 nm LED can control *Salmonella* on cooked chicken [10]. Blue LEDs were also applied to lower the risk of *Listeria monocytogenes* and *Salmonella* by eliminating or inhibiting the bacterial growth on cantaloupes during transport and storage [11]. Ref. [11] found that a 405 nm LED effectively controlled *L. monocytogenes* during biofilm formation and can induce sensitivity to sanitizer treatment of cells in pre-formed biofilms. These studies suggest that LEDs have the potential for application to inactivate foodborne pathogens. However, the effect of 405 nm LED light on stress resistance of *S.* Typhimurium has rarely been reported.

The objective of this research was to evaluate the effect of 405 nm LED-illumination on the survival of *S.* Typhimurium under thermal, osmotic or oxidative stress, or in simulated gastric fluid or bile salts. Furthermore, RT-PCR was performed to investigate the effects of illumination on the expression of environmental tolerance-related genes, to help assess mechanisms of the outcomes of the illumination.

## 2. Materials and Methods

### 2.1. Bacterial Strain and Culture Conditions

*S*. Typhimurium SL1344 (DSM 24522) was purchased from the German Collection of Microorganisms and Cell Cultures (DSMZ, Braunschweig, Germany). Before each experiment, a fresh overnight culture was prepared by incubation at 37 °C for 12 h in 30 mL Tryptone soya broth (TSB, Land Bridge, Beijing, China) with shaking (130 rpm).

### 2.2. Bacterial Inoculation and Preparation of Contaminated Beef Samples

*S*. Typhimurium SL1344 culture was centrifuged at 8000× *g* for 5 (4 °C) and washed with sterile phosphate-buffered saline (PBS; pH 7.2). Then, the bacteria were resuspended in PBS and diluted to approximately 10^9^ colony-forming units [CFU]/mL before each assay.

Beef was purchased from a local market in Yangling, China, and was carefully cut into rectangular slices of approximately 5 g (approx. 3 cm × 5 cm × 3 mm) using a sterile knife in a sterile environment. Before inoculation with *S*. Typhimurium, the beef was exposed to 0.02% (*v/v*) NaClO (Xilong Scientific Co., Ltd., Shantou, Guangdong, China) for 10 min to reduce bacteria that were already present. After that, the sliced beef was put into sterile water twice for 10 min to wash away residual NaClO, and was then air-dried at room temperature for 10 min. The total number of background bacteria in sliced beef above was controlled to be less than 10^3^ CFU/cm^2^. In addition, no *Salmonella* was detected in beef after 0.02% NaClO treatment (the detection limit was 3.33 CFU/mL). Fifty microliters aliquot of *S*. Typhimurium suspension was seeded, divided into 10 spots on each beef slice’s surface to a final concentration of around 10^7^ CFU/cm^2^.

### 2.3. LED Illumination Device

The LED illumination device was set up with 405 nm LEDs (Shenzhen Boya Technology Co., Ltd., Shenzhen, China) surrounded by an acrylonitrile butadiene styrene housing to block the external light during illumination. A cooling fan and heat sink were attached to lower the heat generated by LED. A 5-Ω resistor was used to protect the LEDs, as previously described [12]. A 5-g beef sample, prepared as described in Section 2.2, was put into a sterile Petri dish (90 mm) without lid. The distance between the LED source and the Petri dish was 4.5 cm to ensure that the suspension can by fully illuminated during the LED illumination.

The dose of LED illumination was calculated by the formula as follows:

*E* = *Pt*,

where *E* is the dose (energy density) in J/cm^2^, *P* is the irradiance (power density) in mW/cm^2^, and *t* is the time in seconds [13].

The irradiance of the 405-nm LEDs measured using a LED radiometer (Linshang, Shenzhen, China) was 18.9 ± 1.4 mW/cm^2^. The temperature of the surface of a beef slice was measured every minute for 480 min (8 h) during LED illumination by using a thermocouple thermometer (Everett, WA, USA).

### 2.4. The Inactivation Effect of 405-nm LED on S. Typhimurium SL1344 in PBS and on Beef Slices

#### 2.4.1. Time-Kill Assay in PBS

*S*. Typhimurium SL1344 suspension was prepared as described in Section 2.2 and diluted with PBS to approximately 7.0 log CFU/mL. Then, the 15 mL of bacterial suspension was aspirated into a sterile Petri dish (d = 90 mm) and was illuminated or non-illuminated by the 405 nm LED for 8 h (total dose 544 J/cm^2^) at 4 °C and 5.4 °C, respectively. At sampling time points 0, 2, 4, 6, and 8 h, the illuminated and non-illuminated samples were collected, 10-fold serial diluted in PBS, and plated on Tryptone Soya Agar (TSA; Land Bridge, Beijing, China). The samples were incubated for 24 h at 37 °C before colony enumeration.

#### 2.4.2. Time-Kill Assay in Beef Slices

Contaminated beef slices, prepared as described in Section 2.2, were placed in a sterile Petri dish. The sliced beef in the illuminated group was illuminated by LED light for 8 h (total dose 544 J/cm^2^) at 4 °C. Non-illuminated beef slices were placed at 7 °C in the dark as controls. At each sampling time (0, 2, 4, 6, and 8 h), illuminated or non-illuminated beef slices were transferred into a meat grinder (Joyoung, Jinan, Shandong, China) containing 45 mL of 0.1% (*w/v*) buffered peptone water (BPW, LandBridge, Beijing, China) and were homogenized for 2 min. After homogenization, the samples were serially diluted with sterile PBS, and plated on xylose-lysine-deoxycholate (XLD; LandBridge, Beijing, China)-agar plates. The plates were incubated for 24 h at 37 °C before colony enumeration. The population of surviving bacteria was expressed in log CFU/cm^2^.

### 2.5. Determination of the Resistance of S. Typhimurium in Beef Slices to Environmental Stress

In this study, we assumed that beef is stored and sold in supermarkets and fresh-food stores that are open for 8 h per day; thus, LED illumination for 8 h (480 min) was chosen as the treatment time to investigate the effect of LED illumination on the resistance of *S*. Typhimurium to environmental stresses. To make the initial concentration of bacteria in each group consistent in the experiment after LED illumination, the bacterial solution of the control group was diluted by 10 times and was inoculated on beef before LED illumination. To determine resistance to environmental stress conditions, a beef slice contaminated with *S.* Typhimurium SL1344 was treated with the 405 nm LED apparatus for 8 h at 4 °C. The non-illuminated controls were incubated at 7 °C for 8 h. After that, each sample was added into 45 mL of 0.1% BPW and was homogenized for 2 min.

#### 2.5.1. Resistance of *S*. Typhimurium to Thermal Stress

The homogenized samples in Section 2.5 were divided into 1.5 mL sterile EP tubes and placed in a dry thermostatic metal bath (BL150-1A, Bilang, Shanghai, China) at 50, 55, 60, and 65 °C, respectively. The samples were heated to the target temperature (50, 55, 60, and 65 °C) at the geometrical center in the bath (which needed 2.5, 3, 3 and 3.5 min). When the target temperature was reached, the timing started immediately. After heating, the samples were cooled in an ice-water bath immediately, the samples were serially diluted with sterile PBS and plated onto XLD-agar for 24 h at 37 °C for bacteria counting.

#### 2.5.2. Resistance of *S*. Typhimurium to Osmotic Pressure

NaCl solutions were used to simulate the environmental osmotic pressure faced by bacteria [14]. Homogenized beef samples that were illuminated or non-illuminated by 405 nm LEDs were transferred into 10-mL tubes containing 5 mL NaCl (Xilong Scientific Co., Ltd.) solution (5% or 10% [*v/v*]), then each sample was incubated at 25 °C. After 0, 30, 60, 90, and 120 min, samples were collected and diluted with sterile PBS, and were plated onto XLD-agar for 24 h at 37 °C for colony enumeration (The detection limit is 3.33 CFU/mL).

#### 2.5.3. Resistance of *S*. Typhimurium to Oxidative Stress

Homogenized beef samples that were illuminated or non-illuminated by 405 nm LEDs were transferred into 0.01% (*v/v*) H_2_O_2_ (Xilong Scientific Co., Ltd.) solution; then, the cultures were incubated at 25 °C. The homogenized beef samples were collected from each tube at 5, 10, 20, 30, and 40 min and were serially diluted using sterile PBS. The samples were plated on XLD-agar at 37 °C for 24 h for colony enumeration.

#### 2.5.4. Simulated Gastric Fluid-Resistance of *S.* Typhimurium

The simulated gastric fluid (SGF)-resistance of *S.* Typhimurium was determined by subjecting the bacteria to SGF (pH 2.0 or 3.0) [15]. SGF consisted of 8.3 g/L proteose peptone, 0.6 g/L KH_2_PO_4_ (Xilong Scientific Co., Ltd.), 2.05 g/L NaCl, 0.37 g/L KCl (Xilong Scientific Co., Ltd.), 0.11 g/L CaCl_2_ (Xilong Scientific Co., Ltd.), 0.05 g/L oxgall (Sigma Aldrich, St. Louis, MO, USA), 1 g/L lysozyme (Solarbio, Beijing, China), and 13.3/L mg pepsin (1:3000; Solarbio, Beijing, China). Except for the oxgall, lysozyme and pepsin (sterilized by 0.25-μm filters), all the components were dissolved in deionized water and autoclaved. The solution was adjusted to pH 2 or 3, respectively, with 6.0 M HCl. Homogenized beef samples from the 405-nm LED illuminated and non-illuminated groups were transferred into SGF at a ratio of 1:9 (homogenized sample: SGF solution) and incubated with shaking (130 rpm) at 37 °C. At sampling times, each sample was serially diluted in PBS and plated on XLD-agar at 37 °C for 24 h for colony enumeration.

#### 2.5.5. Bile Salt-Resistance of *S.* Typhimurium

The resistance of *S*. Typhimurium to bile salts was investigated as in a previous study [3]. Homogenized beef samples were mixed with pre-prepared 1%, 2% and 3% (*w/v*) bile salts (Sigma) solutions, respectively. Then, each mixture was placed at 37 °C with agitation (45 rpm) for 0, 5, 10, 20, 40, and 60 min. After incubation, the samples were serially diluted using sterile PBS, and plated on XLD-agar at 37 °C for 24 h for colony enumeration.

### 2.6. Effect of 405 nm LED-Illumination on Gene Transcription in S. Typhimurium

The *S*. Typhimurium SL1344 suspension was illuminated by 405 nm LED apparatus at 4 °C for 240 min, and non-illuminated (control) cells were incubated at 7 °C for 240 min. After incubation, each sample was centrifuged (5000× *g*, 5 min, 4 °C) and resuspended in PBS. Total RNA was extracted using a Bacterial RNA Extraction Kit (Tiangen, Beijing, China) according to the manufacturer’s instructions. RNA integrity and concentration were measured using a spectrophotometer (Nano-200; Aosheng Instrument Co., Ltd., Hangzhou, China). The RNA was then immediately reverse-transcribed to complementary DNA using the Takara PrimeScript RT Reagent Kit (Takara, Kyoto, Japan). Real-time polymerase chain reaction was conducted with the IQ_5_ system (Bio-Rad Laboratories, Hercules, CA, USA). The 16S rRNA gene was used to normalize the gene expression levels. The sequences of the primers are listed in Table S1. The transcription of target genes was analyzed with the −1/2^–ΔΔ*Ct*^ method [16].

### 2.7. Statistical Analysis

All experiments were proceeded at least three times. The data are expressed as mean ± standard deviation and analyzed using SPSS 19.0 software (SPSS Inc., Chicago, IL, USA). The significant difference used the least significant difference method. The differences between the groups were considered significant and extremely significant at *p* < 0.05 and *p* < 0.01, respectively.

## 3. Results

### 3.1. The Temperature Profile during 405 nm LED-Illumination

**Figure 1:**
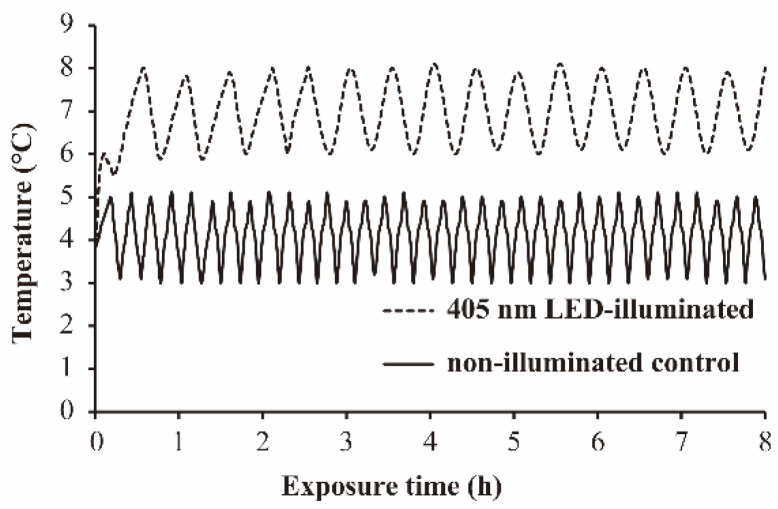
Temperature profile on beef slice during 405 nm LED-irradiation at 4 °C.

The temperature profile on the surface of the beef slices and in PBS during 405 nm LED-illumination at 4 °C is shown in Figure 1 and Figure S1 respectively. The temperature of PBS suspension increased by 1.4 °C during LED-illumination (Figure S1). The temperature of the *S*. Typhimurium-contaminated beef slice surface increased to 7 °C (mean temperature) during LED-illumination. During our test time (8 h), the temperature profile of the bacterial suspension and the beef slice surface showed regular fluctuations (Figure 1 and Figure S1). On the basis of these findings, non-illuminated bacterial suspension and non-illuminated beef slices were placed at 5.4 °C and 7 °C, respectively, to compensate for the temperature increase of the illuminated samples during the illumination.

### 3.2. Survival of S. Typhimurium following 405 nm LED Treatment

**Figure 2:**
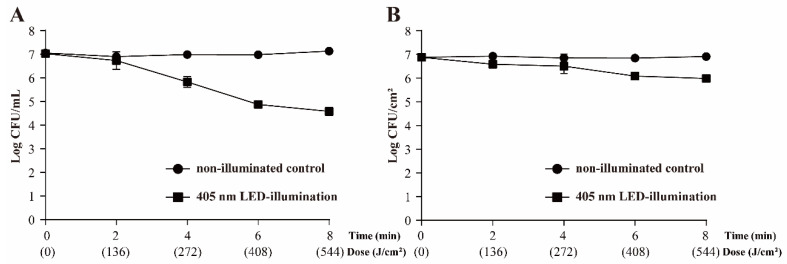
Effect of 405 nm LED-illumination on *Salmonella* Typhimurium in phosphate-buffered saline (PBS) (**A**) and on beef slices (**B**) at 4 °C.

Time–kill curves are shown in Figure 2. The 405 nm LED-illumination led to 1.19 and 2.44 log CFU/mL decreases of the cell population at 4 °C in PBS after treatment for 4 h (total dose 273 J/cm^2^) and 8 h (total dose 546 J/cm^2^), respectively (Figure 2A). The number of *S*. Typhimurium cells in the non-illuminated control remained at the original level (approximately 7.13 log CFU/mL).

In Figure 2B, the initial population of *S*. Typhimurium on the beef slice surface was approximately 6.88 log CFU/cm^2^. The population of *S*. Typhimurium cells in the non-illuminated control remained at the original level with 8 h. On beef slices illuminated by the 405-nm LED, the population of *S*. Typhimurium was significantly decreased, to 5.99 ± 0.07 CFU/cm^2^ (*p* < 0.01).

### 3.3. Environmental Stress-Resistance of S. Typhimurium in Sliced Beef

#### 3.3.1. Resistance of *S*. Typhimurium to Heat Stress

**Figure 3:**
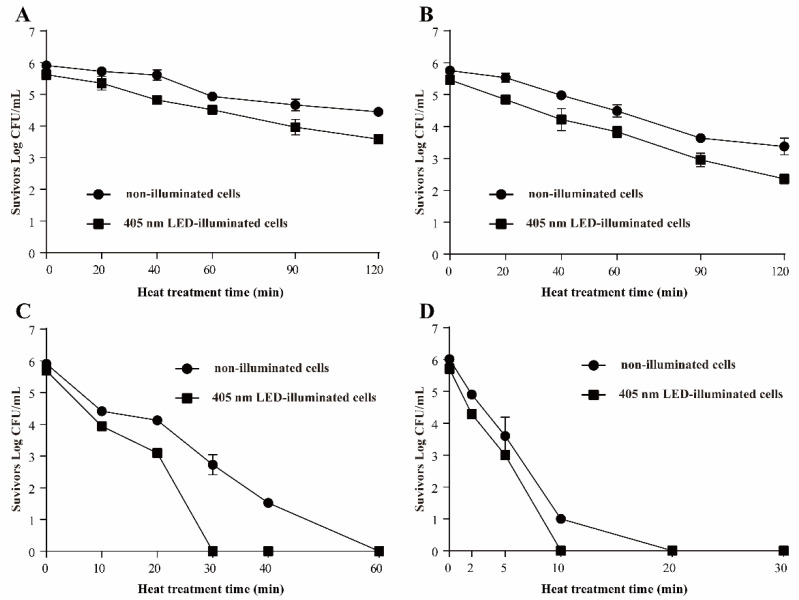
Survival curves at 50 °C (**A**), 55 °C (**B**), 60 °C (**C**), and 65 °C (**D**) for *S*. Typhimurium. (●) non-illuminated cells, (■) 405 nm LED-illuminated cells.

The effects of 405 nm LED-illumination on the heat resistance of *S.* Typhimurium are shown in Figure 3. The initial populations of non-illuminated cells and LED illuminated cells were 5.75 ± 0.08 and 5.46 ± 0.13 log CFU/mL, respectively. On incubation at 50 °C, the population of *S*. Typhimurium in the non-illuminated group decreased by approximately 1.47 log CFU/mL within 120 min, and the number of 405-nm illuminated cells decreased by approximately 2.03 log CFU/mL (Figure 3A). On incubation at 55 °C, after 120 min, the number of illuminated *S*. Typhimurium was respectively decreased to 3.38 ± 0.26 and 2.36 ± 0.15 log CFU/mL (Figure 3B). At 60 °C, a decrease of approximately 3.18 log CFU/mL was observed after 30 min in the control (non-illuminated) sample, but the cell population in the LED illuminated sample was decreased to an undetectable level (3.33 CFU/mL) at 30 min (Figure 3C). At 65 °C (Figure 3D), the LED treated *S*. Typhimurium population dropped below the detectable level (3.33 CFU/mL) within 10 min, compared with 20 min for the non-illuminated controls.

#### 3.3.2. Resistance of *S*. Typhimurium to Osmotic Pressure

**Figure 4:**
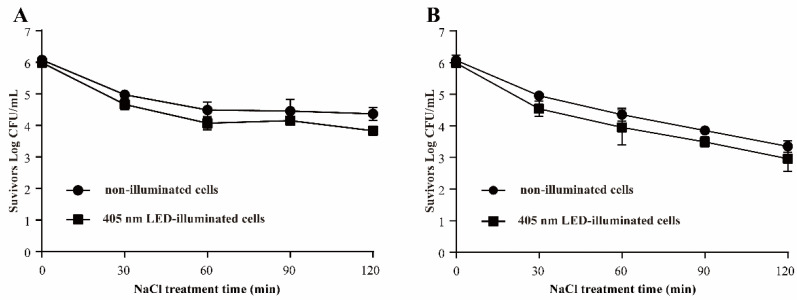
Survival of *S*. Typhimurium in 5% (**A**) and 10% NaCl (**B**). (●) non-illuminated cells, (■) 405 nm LED-illuminated cells.

In Figure 4, when *S*. Typhimurium was exposed to 5% NaCl for 120 min, a decrease of 2.17 log CFU/mL was observed in the 405 nm LED-illuminated samples, compared with 1.71 log CFU/mL for the non-illuminated controls. After exposure to 10% NaCl for 120 min, the decreases in the numbers of LED treated cells and control cells were 2.93 and 2.70 log CFU/mL, respectively.

#### 3.3.3. Resistance of *S*. Typhimurium to Oxidative Stress

**Figure 5:**
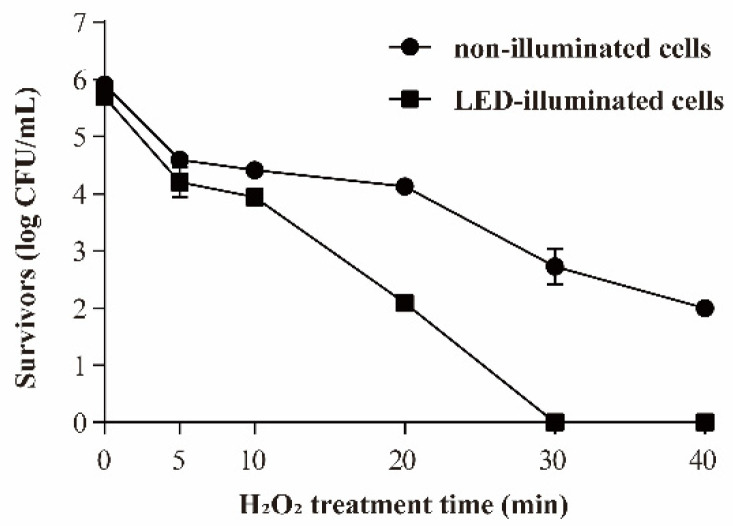
Survival of *S*. Typhimurium in 0.01% H_2_O_2_. (●) non-illuminated cells, (■) 405 nm LED-illuminated cells.

The effect of 405 nm LED-illumination on the oxidative stress resistance of *S.* Typhimurium is shown in Figure 5. The initial populations of bacteria were 5.91 ± 0.11 and 5.69 ± 0.0.9 log CFU/mL in the LED-illuminated and non-illuminated control groups, respectively. When the samples were exposed to 0.01% (*v/v*) H_2_O_2_, the number of non-illuminated *S*. Typhimurium survivors decreased to around 2.00 log CFU/mL within 40 min, but the cell population in the LED-illuminated sample decreased to an undetectable level (3.33 CFU/mL) within 30 min.

#### 3.3.4. Resistance of *S*. Typhimurium to SGF

**Figure 6:**
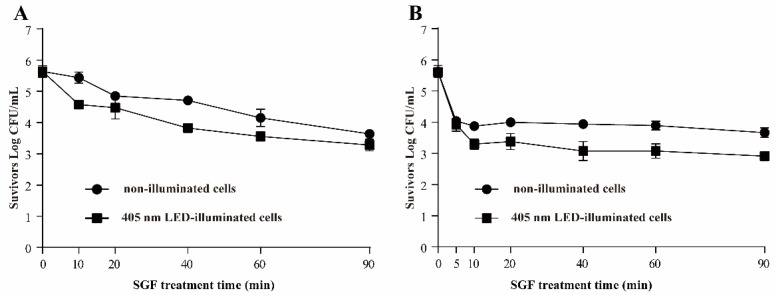
Survival of *S*. Typhimurium in simulated gastric fluid (SGF) at pH 3 (**A**) and pH 2 (**B**). (●) non-illuminated cells, (■) 405 nm LED-illuminated cells.

The effects of 405 nm LED-illumination on the resistance of *S.* Typhimurium to SGF at pH 2 and 3 are shown in Figure 6. LED treated *S*. Typhimurium exhibited a diminished ability to survive acid stress compared with the non-illuminated controls. The population of illuminated bacteria decreased faster than the non-illuminated cells when *S*. Typhimurium were exposed to SGF at pH 3: the population of LED treated cells was decreased by 2.36 log CFU/mL within 90 min, while the decrease in control group was around 2.00 log CFU/mL (Figure 6A). When *S*. Typhimurium were exposed to SGF at pH 2, the bacterial populations decreased markedly within the first 10 min in both the treated and control samples, and no further decrease was observed over the next 80 min (Figure 6B). The population of LED treated cells was decreased by 2.70 log CFU/mL, and the number of non-illuminated cells decreased by 1.96 log CFU/mL.

#### 3.3.5. Resistance of *S*. Typhimurium to Bile Salts

**Figure 7:**
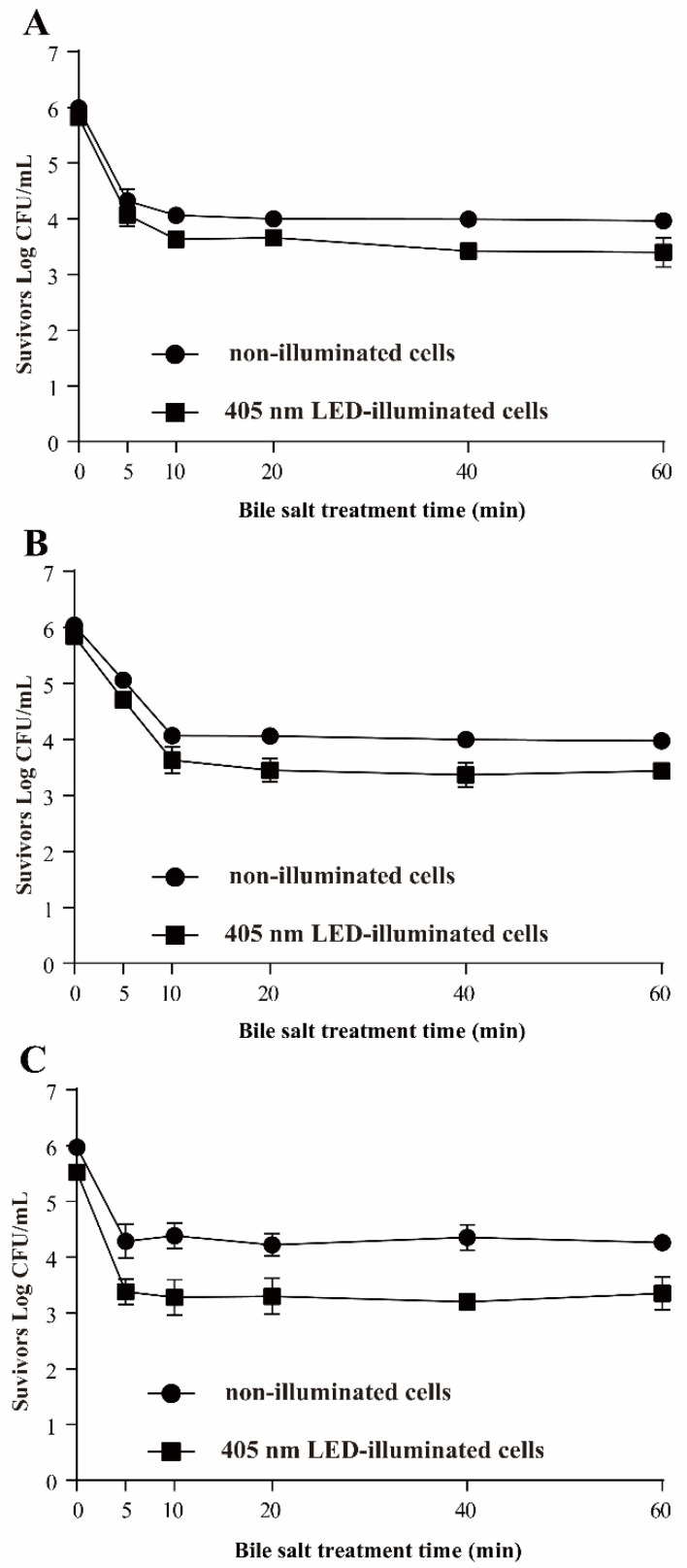
Survival curves of *S*. Typhimurium in 1% (**A**), 2% (**B**), and 3% (**C**) bile salts solution. (●) non-illuminated cells, (■) 405 nm LED-illuminated cells.

Figure 7 shows the population of LED-illuminated and non-illuminated *S*. Typhimurium after treatment with different concentrations of bile salts. The initial populations of non-illuminated cells and LED illuminated cells were 5.99 ± 0.07 and 5.82 ± 0.08 log CFU/mL, respectively. After exposure to 1.0% bile salts for 60 min, the population of each group was significantly decreased, by 2.02 and 2.42 log CFU/mL (*p* < 0.01), respectively (Figure 7A). A decrease of around 2.07 log CFU/mL was observed for the control after exposure to 2.0% bile salts for 60 min, and the cell population of the LED illuminated sample was decreased by 2.41 log CFU/mL (Figure 7B). Similar trends were observed for 3.0% bile salts treatment: the population of LED treated *S*. Typhimurium decreased to 3.52 ± 0.30 log CFU/mL, and that of non-illuminated *S*. Typhimurium to 4.26 ± 0.12 log CFU/mL, within 60 min (Figure 7C). Notably, the major part of the decrease in all the bacterial populations occurred within the first 5–10 min of bile salts treatment.

### 3.4. Effects of 405 nm LED Illumination on Gene Transcription of S. Typhimurium

**Figure 8:**
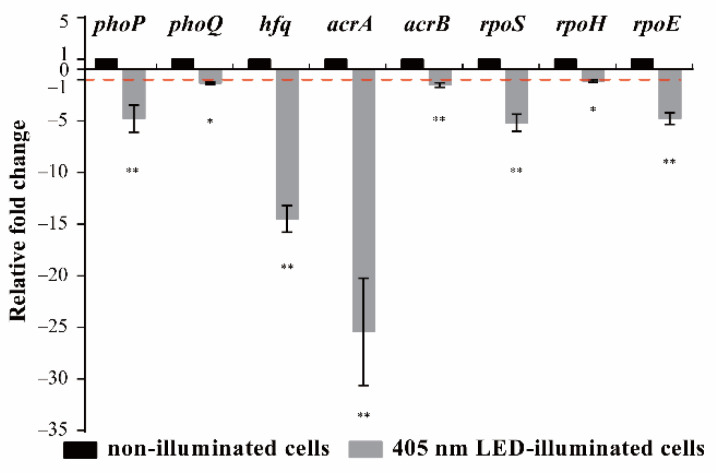
Transcription levels of *S*. Typhimurium environmental tolerance-associated genes. * *p* < 0.05, ** *p* < 0.01 vs. non-illuminated cells.

In Figure 8 and Table S1, LED illumination significantly affected the transcription of environmental stress-related genes in *S*. Typhimurium. The transcription of *rpoS* (critical for regulating the general stress response) and *rpoE* (critical for regulating resistance to oxidative stress) was significantly downregulated by 405 nm LED illumination compared with non-illuminated controls (*p* < 0.05). The transcription levels of *phpP* and *phoQ* (critical for regulating resistance to acid stress) were, respectively, 0.22 and 0.74 times lower in illuminated cells compared with non-illuminated cells; Furthermore, the transcription levels of *hfq* (general stress response) and *acrA* (oxidative stress) in non-illuminated cells were 14.49 and 25.43 times higher than those in illuminated cells, respectively, and the levels of *acrB* and *rpoH* (oxidative stress) were 1.81 and 1.51 times higher.

## 4. Discussion

*Salmonella* cells in contaminated food may be faced with various stresses during food processing, storge and transportation [3]. The main stresses imposed on *Salmonella* in food processing include osmotic stress (salt, preservation, and flavor enhancers), thermal stress (pre-cooking and cooking), and oxidative stress (disinfection) [17]. A previous study showed that *Salmonella* have evolved to survive in naturally stressful conditions such as high osmolarity, extreme temperatures, and low pHs [8]. After suffering these environmental stresses, surviving *Salmonella* cells that contaminate food and thus are ingested into the human body will encounter the stomach (acid stress) and intestine (bile salts) in sequence [3]. The environmental resistances of *Salmonella* increase the ability to survive in the food processing chain and increase the risk of human infection and the difficulty of removing the bacteria. Therefore, lowering the tolerance of *Salmonella* to environmental stress is important for preventing contamination in food chains and infections associated with *Salmonella*. LEDs are a novel physical sterilization method that were reported to have antibacterial effects against both Gram-negative and Gram-positive bacteria [10,18]. However, the effect of 405 ± 5 nm LED on the environmental stress tolerance of *Salmonella* has not been reported. In this study, the effects of 405 nm LED illumination on the resistance of *S.* Typhimurium in beef slices at storage temperature to diverse environmental stresses were investigated.

Heat treatment is commonly used for the decontamination of foodborne pathogens [19]. A previous study reported that *S*. Typhimurium and *S*. Enteritidis are more tolerant of stress conditions such as heat, acid and osmotic stress than other *Salmonella* serovars [17]. *S*. Typhimurium isolated from milk or humans shows thermal resistance (reported *D*-values of 0.11 min at 62.8 °C and 24 min at 51.8 °C) [20]. In the present study, the population of LED-illuminated cells showed fewer survivors than the non-illuminated controls after treatment at 50, 55, 60, or 65 °C (Figure 3A–D), which confirmed that the bacteria cells illuminated by LED were more susceptible to thermal stress than non-illuminated bacteria. Cell membrane integrity plays a key role in protecting microorganisms against various stress conditions. Decreased heat tolerance may occur because of the lowered melting point of unsaturated fatty acids within the cell membrane [21]. Moreover, the induction of RpoH can guide the transcription of molecular chaperones and protease-encoding genes, which are involved in the folding or degradation of heat-damaged polypeptides in the cytoplasm. This is a protective mechanism that may make bacteria more resistant to heat treatment [22]. The results of RT-PCR in this study showed that the mRNA level of *rpoH* was downregulated by LED treatment (Figure 8). Therefore, we speculated that the decrease of the tolerance to heat stress of LED treated cells may be due to membrane injury and downregulation of the transcription of *rpoH*.

Salt is an important ingredient used in food processing and adds flavor. NaCl is always used as the salting source, and it results in high osmotic pressure on bacteria in food, which can help to control foodborne pathogens [19]. The resistance of bacteria to osmotic stress is due to their ability to accumulate compatible solutes to balance osmotic changes [23]. In this present study, *S*. Typhimurium was exposed to NaCl at 5% and 10% to evaluate the changes of its resistance of osmotic pressure illuminated by 405 nm LED. The results show that the 405 nm LED treatment did not decrease the tolerance of *S*. Typhimurium to osmotic pressure significantly (Figure 4). However, after being exposed to 405 nm LED, the percentage of *L. monocytogenes* and *B*. *cereus* sensitivity to NaCl reached more than 90% after 3 h illumination [18]. A previous study reported that *L*. *monocytogenes* and *S*. *aureus* cells in TSB were sensitive to NaCl after treatment of 461 and 521 nm LED [24]. Cells with a dam membrane might be incapable of recovering in a medium containing certain concentrations of NaCl due to a loss of osmotic functionality of cytoplasmic membranes [18]. In a previous study, LED-illumination caused the loss of cell membrane integrity in *E. coli* O157:H7, *S.* Typhimurium and *S.* sonnei [25].

*Salmonella* is exposed to oxidation stress as they pass through both host and non-host environments [17]. Hydrogen peroxide (H_2_O_2_) is commonly used as a sanitizer for foodborne pathogens’ elimination during food processing [26]. H_2_O_2_ indirectly generates oxidative species which can cause oxidation damage to some cellular components such as DNA, proteins, and cell membranes [8]. In this research, the LED-illuminated cells were more sensitive to the oxidative environment (0.03% H_2_O_2_) and the population of survivors was decreased to an undetectable level within 30 min (Figure 5). Álvarez-Ordóñez et al. [27] confirmed that the growth of *S.* Typhimurium in the presence of organic acids resulted in an increased sensitivity to H_2_O_2_. The ArcAB two-component system is a regulating system that responds to oxidation stress [28]. ArcA plays an important role in the resistance of oxidation stress of *S*. Typhimurium, and ArcB can transfer the phosphate group to the cytoplasmic response regulator ArcA to response the oxygen [29]. The sigma factors, σE (RpoE) and σH (RpoH), also promote antioxidant defenses by enhancing σS levels [30]. In this study, the transcription levels of *acrA*, *acrB*, *rpoE* and *rpoH* were reduced by LED-illumination (Figure 8). Therefore, the decrease of the oxidative resistance of *S.* Typhimurium induced by 405-nm LED treatment may be related to its effect on the function of the ArcAB two-component system and sigma factors.

Acidic conditions are the main stress that food-borne pathogens encounter when they come into contact with stomach contents. It is characterized by a low pH (approximately pH 2) and is considered to be another barrier against food-borne pathogens. [31]. A previous study reported that membrane composition allows pathogenic bacteria to adapt to acidic conditions [32]. Spector and Kenyon [17] reported that *Salmonella* can survive exposure to the normally lethal pH of 2.5. In the present study, simulated gastric fluids (pH 2 or 3) were used. The acid-sensitivity of *S*. Typhimurium was significantly enhanced (*p* < 0.01) by 405 nm LED-illumination compared with cells without illumination (Figure 6). Similarly, resveratrol lowered the resistance to acid stress of *Staphylococcus aureus* and *L. monocytogenes* [33]. Moreover, Lehrke et al. [34] reported that nisin and green tea extracts increased the susceptibility of *L. innocua* to acid stress: after being treated with nisin (30 IU/mL) or green tea extract (5390 mg/L) for 48 h and treatment in an acidic environment (pH 4.0) for 1 h, *L. innocua* decreased by 2.7 and 1.4 log CFU/mL, respectively, while the number of untreated bacteria only decreased by 0.2 log CFU/mL. It has been suggested that H^+^ sensing systems, such as PhoP/Q on the membrane of *Salmonella*, may contribute to enhanced acid tolerance [35]. Our RT-PCR results revealed that the transcription of *phoP* and *phoQ* was downregulated by LED illumination. Therefore, we hypothesize that LED illumination affects the acid tolerance of *S.* Typhimurium by damaging the cell membrane and by regulating signal transduction via PhoP/Q.

Foodborne pathogens must survive not only stomach acidity but also bile in the intestine. Bile salts, produced from cholesterol by the liver and secreted into bile, are detergent-like compounds that aid in the digestion and dispersion of dietary fats in the intestine. They are also bactericidal [36]; the antimicrobial activity of bile is primarily the result of the bile salts [17]. Under normal physiological conditions, the concentration gradient of bile salt in the human intestine is between 2% and 0.05% [37]. Enteric organisms, such as *S.* Typhimurium, are resistant to bile salts at concentrations above even those found in the small intestine [36]. In the current study, the resistance of LED-illuminated *S*. Typhimurium to bile-salts (1%, 2%, and 3%) was decreased significantly (*p* < 0.01) compared with non-illuminated controls. In a previous study [14], the population of *L. monocytogenes* illuminated by LEDs for 120 min was decreased by about 1.7 log CFU/mL when exposed to bile salt at 2%, while the non-illuminated controls maintained their initial population. Thus, LED-illumination can decrease the ability of pathogens to survive in bile salts solutions. Previous studies reported that bile in the intestines disorganizes the structure of the bacterial membrane and triggers DNA damage (Hsiao et al., 2010), and that multidrug efflux pumps can remove bile that gets through the outer membrane. For example, the AcrAB pump of *S.* Typhimurium is absolutely required for bile resistance [38]. Therefore, the increased sensitivity of LED treated *S*. Typhimurium to bile salts might be due to damage to the cell structure and DNA, as well as downregulation of the expression of the AcrAB pump (Figure 8).

RpoS is a sigma factor that alters gene expression profiles in response to a variety of stresses such as acidic pH, high osmolarity and temperature shock [39]. Sittka et al. [40] reported that Hfq was shown to promote efficient translation of *rpoS* mRNA in *Salmonella.* In the present study, RT-PCR analysis showed that the transcription levels of *hfq* and *rpoS* were downregulated in illuminated cells compared with non-illuminated controls. These findings suggested that the increased sensitivity of LED treated *S*. Typhimurium to environmental stresses was probably due to regulating the transcription levels of environmental stress-related genes.

## 5. Conclusions

The results of this research confirm that 405 nm LED-illumination decreases the survival rate of *S*. Typhimurium in beef slices and lowers the resistance of *S*. Typhimurium to heat (50–65 °C), oxidative stress (0.01% H_2_O_2_), acid stress (SGF at pH 2 or 3), and bile salts (1–3%). LED illumination also downregulated the transcription of genes related to acid, and heat stress-resistance. Therefore, LED-illumination is an effective strategy to decrease the environmental tolerance of *S.* Typhimurium. Combined with its low energy consumption, high safety, and ability to be easily combined with production, processing, storage and consumption, treatment with LED-illumination has the potential to be applied in food processing, storage and transportation to control *Salmonella* contamination and decrease the infection risk associated with Salmonella.

## Data Availability

The data presented in this study are available on request from the corresponding authors.

## Supplementary Materials

The following are available online at https://www.mdpi.com/article/10.3390/foods11020136/s1, Figure S1: Temperature profile in phosphate-buffered saline during 405 nm LED-illumination at 4 °C. Table S1: Primers of environmental tolerance-associated genes of *S*. Typhimurium SL1344.
