# Supplementary material for: Effects of 405 ± 5-nm LED Illumination on Environmental Stress Tolerance of Salmonella Typhimurium in Sliced Beef
Source: Foods. 2022 Jan 6;11(2):136. doi: 10.3390/foods11020136 (PMC8774786; doi:10.3390/foods11020136)
Supplement: Supplementary file 1 [file foods-11-00136-s001.zip › foods-1508592-supplementary.pdf]

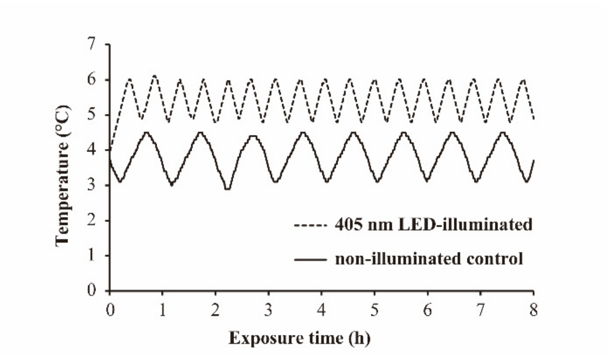

Figure S1. Temperature profile in phosphate-buffered saline during 405 nm LED-illumination at 4 °C.

Table S1. Primers of environmental tolerance-associated genes of *S.* Typhimurium SL1344.

Target gene	Sequence of primers (5′–3′)	Reference
16S rRNA	F, AGGCCTTCGGGTTGTAAAGT<br>R, GTTAGCCGGTGCTTCTTCTG	[1]
phoP	F, GAAGGCTGGCAGGATAAA<br>R, CGGATAAGCGTTTCCATAA	This study
phoQ	F, ACAAATACCGCACGACCC<br>R, CCGGCTGATCTGTTCCAG	This study
hfq	F, TAAGCTGCAAGGTCAAATC<br>R, GACGGGACAACAGTAGAAAT	This study
acrA	F, CGGTCGTATTGGTAAGTCATC<br>R, GGTAACGCCCTGTTGTGGA	This study
acrB	F, TCTATCCCGTTCTCCGTAATG<br>R, GAACTGATAACCAGCGGCATA	This study
rpoS	F, CAGCCGTATGCTTCGTCTCA<br>R, TTTTCATCGGCCAGGATGTC	This study
rpoH	F, TTATCGGCTGACGAGGAG<br>R, TGTACGGCGAAAGAAACC	This study
rpoE	F, AAGTGGCGAGTCTGGTTT<br>R, CTCCCGTAAGGTGATTGC	This study

1. Lamas, A.;Regal P.;Vázquez B.;Miranda J. M.;Cepeda A.;Franco C. M. Influence of milk, chicken residues and oxygen levels on biofilm formation on stainless steel, gene expression and small RNAs in Salmonella enterica. *Food Control*, **2018**, *90*: 1-9.
